# Charity Midwifery in London

**Published:** 1898-09-24

**Authors:** 


### Charity Midwifery in London.

When young men and fair maidens plight their troth it is probable that they think but little of the consequences. Perhaps if they did, and if they knew the risks of matrimony, young women would pause on the brink and hesitate more than they do in entering on an adventure which, if a family is to follow in due course, must entail upon them a not inconsiderable chance of death. What this chance is, was discussed at the meeting of the British Medical Association at Edinburgh, and the result of the discussion was to suggest that it is a good deal higher than the insurance companies have hitherto calculated it to be.

It is a curious commentary upon the deficiencies of our registration system that it seems impossible to make out with anything like certainty in what proportion of cases childbirth is followed by death, although both births and deaths are events which have to be registered in accordance with Act of Parliament, and in most instances are registered by the same official. What seems, however, to have been clearly made out by Dr. Boxall's well-known investigations into the returns of the Registrar-General is that, taking a considerable number of years, the registered mortality of childbirth works out to nearly one death to every 200 births. It is well recognised, however, that there are many chances of error in regard to these returns, all of which tend to make them under-estimate the mortality. Others inquirers, indeed, have made out the mortality of childbirth to be much higher than this, and Dr. John Playfair and Mr. Wallace have recently shown that the record of the Edinburgh Royal Maternity and Simpson Memorial Hospital for ten years and a half give a rate of one death in 115 births.

Mrs. Garrett Anderson maintains that such a death-rate is far too high, and she points to the records of certain charities in London and in Dublin as showing how very much lower a rate is possible even among patients attended in their own homes. The figures are very large, and, taking them in the gross, the results are striking. Taking nine maternity charities and the maternity departments of six general hospitals, she shows that among the 71,122 deliveries conducted by them 151 deaths took place, giving a death-rate of one in 471, and she concludes that if such results can be obtained in charity midwifery, even when carried on in the homes of the poor, the total average puerperal mortality is far higher than it ought to be. With this we agree, although we hardly feel prepared to admit that these returns express the full mortality of the charities in question. If they do, however, and if they are to be taken as Mrs. Garrett Anderson has taken them as expressing the death-rate which is met with in the practice of these various charities, and if, further, this comparatively low mortality among the charities is to be accepted as showing that the general puerperal mortality of the country is too high, another question arises which is of very immediate and urgent importance, for within the group of charities on whose returns Mrs. Anderson depends in her condemnation of the general puerperal death-rate of the country, there are variations in mortality far greater than the difference between her ideal rates and the actual death-rate deduced by Dr. Boxall from the Registrar-General's returns. Thus the death-rate at the Charing Cross Hospital is put down as one in 229·2, while that at St. Bartholomew's is only one in 838·1, and the return from the New Hospital for Women shows a score of 1,163 and "not out"—1,163 cases attended without a death. Here is indeed a great discrepancy, and we think that Mrs. Garrett Anderson may fairly be asked to take the responsibility of her own figures, and to follow her own argument as far as it will go. If her statistics are to be accepted as proving what she brings them to prove, namely, that the general average puerperal mortality of the country is much higher than it ought to be, a comparison of the death-rates at the various charities she mentions proves with even greater force that the midwifery conducted by some of the maternities of London requires a thorough overhauling.

